# Upper Quarter Y Balance test performance: Normative values for healthy youth aged 10 to 17 years

**DOI:** 10.1371/journal.pone.0253144

**Published:** 2021-06-18

**Authors:** Gerrit Schwiertz, Julian Bauer, Thomas Muehlbauer

**Affiliations:** Division of Movement and Training Sciences/Biomechanics of Sport, University of Duisburg-Essen, Essen, Germany; University of Pittsburgh, UNITED STATES

## Abstract

**Background:**

The Upper Quarter Y Balance test (YBT-UQ) is a field test for measuring shoulder mobility/stability. However, there is a lack of information regarding age- and sex-specific reference values for classifying the YBT-UQ performance of children and adolescents.

**Objective:**

The aim was to investigate YBT-UQ performance in youth and to provide age- and sex-specific normative values.

**Method:**

Six hundred and sixty-five persons (325 girls, 340 boys) aged between 10 and 17 years carried out the YBT-UQ test. Following this, maximum reach values, normalised in terms of arm length, were calculated for each arm (i.e., left and right) and reach direction (i.e., medial [MD], inferolateral [IL], superolateral [SL]), and the composite score (CS) per arm. Additionally, percentiles were displayed graphically and in tabular form, distinguished according to age and gender.

**Results:**

In boys, those aged 14–15 years showed a higher achievement (e.g., MD direction) compared with both younger (12–13-year-olds) and older (16–17-year-olds) persons. In girls, differences related to age could only be observed for the IL direction and the CS, where the youngest age group (10–11-year-olds) achieved better results than the older groups. Sex-specific differences to the girls’ advantage could be observed in 12–13-year-olds (i.e., SL and CS), and to the boys’ advantage in 14–15-year-olds (i.e., for all reach directions) and 16–17-year-olds (i.e., IL and SL direction and CS). Further, curvilinear developments were observed with regard to the 10^th^, 50^th^, and 90^th^ percentiles, and were more strongly marked in boys than in girls.

**Conclusions:**

The obtained age- and sex-specific normative values for the YBT-UQ can be used by teachers, coaches, and therapists to classify the level of shoulder mobility/stability among 10–17 year-old children and adolescents.

## Introduction

**Fig 1:**
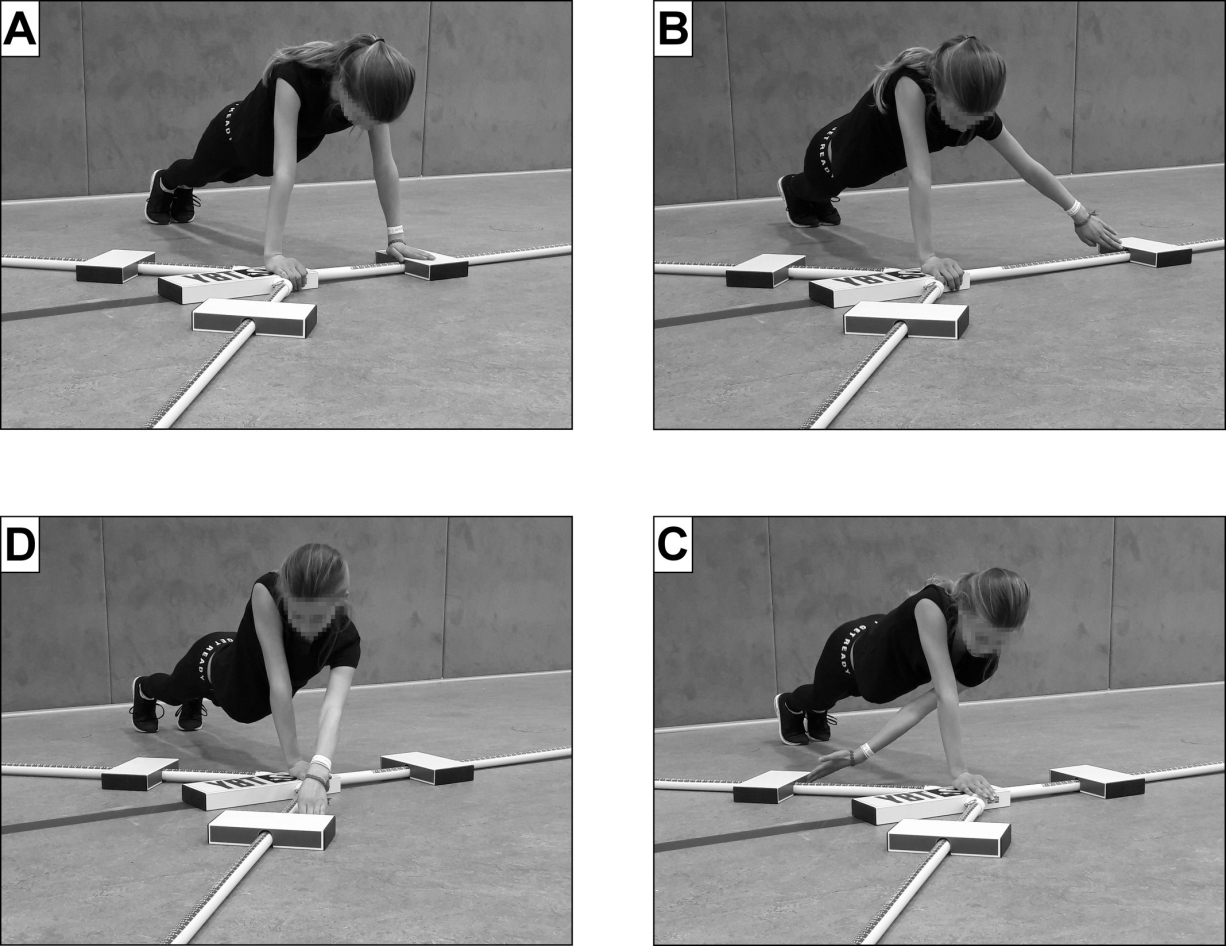
Setup for the assessment of Upper Quarter Y Balance test performance with A) starting position, B) medial, C) inferolateral, and D) superolateral reach directions.

The Upper Quarter Y Balance test (YBT-UQ) is a widely used test procedure carried out in the field to obtain valid and reliable measurements of shoulder mobility/stability in healthy youth [1,2]. Carrying out the YBT-UQ test requires the subject to assume a push-up position on the floor (Fig 1A–1D) supported by one arm and reach as far as possible to the (a) medial (MD), (b) inferolateral (IL), and (c) superolateral (SL) direction with the other arm [3]. In order to enable comparisons between individuals, the absolute reach values achieved (in cm) have to be normalised according to the individual’s arm length (% AL). Although this assessment procedure can reveal performance differences with respect to age (i.e., young vs. old persons) [4], sex (i.e., females vs. males) [5], training status (i.e., untrained vs. trained subjects or athletes with different competition levels) [6,7], previous injuries (e.g., people with and without a history of a shoulder injury) [8], and musculoskeletal injury risk [9], it remains unclear whether the score achieved should be classified as good or poor. However, a classification of the performance level is important, as it enables specifically customised interventions to be derived on the basis of achievement level. For example, one might recommend a programme that boosts motor ability to people achieving poor results, and a programme for talent development to people achieving good ones.

To date there have been relatively few studies [4,10,11] that have created age- and/or sex-specific standard values for the YBT-UQ. Moreover, these studies have been confined to investigations of adults. For example, Borms and Cools [10] investigated male and female athletes (handball, volleyball, tennis) aged between 18 to 50 years. The authors found that the 18–25-year-olds achieved significantly better YBT-UQ results than those aged between 26–33 years or 34–50 years. In addition, they reported significantly better YBT-UQ performance in men compared to women. However, it is questionable to directly transfer these findings to children and adolescents, since their physical development is still continuing [12].

Our aim was therefore to investigate YBT-UQ performance in youth and to provide age- and sex-specific normative values. Thus, we used the YBT-UQ to assess upper quarter mobility/stability in healthy females and males aged 10 to 17 years, statistically compare performance data between ages (i.e., 10-11-, 12-13-, 14-15-, and 16-17-year-olds) and gender, and establish age- and sex-specific percentile values. We hypothesized that YBT-UQ performance improves with increasing age and that differences exist between sexes. From a practical perspective, the generation of age-and sex-specific normative values in young persons is useful to identify individuals for health or talent development programmes.

## Methods

### Participants

Six hundred and sixty-five persons (325 girls, 340 boys) aged between 10 and 17 years took part in the study and were divided into the following four age groups: 10–11-year-olds (*n* = 56), 12–13-year-olds (*n* = 175), 14–15-year-olds (*n =* 218), and 16–17-year-olds (*n =* 216) (Table 1). The subjects were recruited from randomly chosen urban public schools in the Ruhr metropolitan area and exhibit a wide physical activity range. This ranges from only participating in mandatory physical education classes to additional sports club participation (2–3 times per week). Pupils were excluded from study participation if they (1) were outside of the age range 10 to 17 years, (2) had a musculoskeletal, neurological or orthopaedic disorder during the last three months prior to the assessment, (3) had other medical conditions that restricted their ability to execute the YBT-UQ or (4) accomplished the assessment of anthropometric variables or YBT-UQ performance only. Nearly 5–10% of the pupils per class were excluded from study participation because they were not willing to perform the assessment of body mass or the YBT-UQ. Participants’ assent and parents’ written informed consent were obtained prior to the start of the study. The Human Ethics Committee at the University of Duisburg-Essen, Faculty of Educational Sciences approved the study protocol (approval number: TM_28.03.18).

**Table 1:** Characteristics of the study participants (*N* = 665) by age group and sex.

Age group	10–11 years (*N* = 56)		12–13 years (*N* = 175)		14–15 years (*N* = 218)		16–17 years (*N* = 216)	
Sex	f (*n* = 35)	m (*n* = 21)	f (*n* = 91)	m (*n* = 84)	f (*n* = 88)	m (*n* = 130)	f (*n* = 111)	m (*n* = 105)
Body height [cm]	146.9 (9.2)	145.7 (6.7)	160.2 (8.1)	161.3 (11.2)	165.8 (7.4)	177.1 (8.9)	168.0 (8.3)	175.1 (13.1)
Body mass [kg]	39.0 (7.0)	38.0 (6.2)	51.1 (11.6)	49.4 (9.5)	61.7 (11.6)	66.0 (12.3)	64.2 (10.7)	69.4 (13.2)
BMI [kg/m^2^]	17.9 (1.8)	18.1 (2.7)	19.8 (3.4)	18.8 (2.5)	22.3(3.7)	20.9 (2.7)	22.6 (3.1)	22.5 (3.2)
Left arm length [cm]	73.6 (4.8)	74.1 (4.3)	80.6 (4.7)	81.5 (5.4)	83.9 (4.2)	89.7 (5.3)	86.0 (4.7)	89.4 (5.5)
Right arm length [cm]	73.9 (4.6)	74.1 (4.5)	80.6 (5.2)	81.7 (5.4)	84.2 (4.1)	90.1 (5.3)	86.4 (4.5)	89.7 (5.3)

Data are mean values and standard deviations in parentheses. BMI = body mass index; f = female; m = male.

### Testing procedures

The length of the left and the right arm of all participants was determined by measuring the distance in centimetres from the spinal process of the 7^th^ cervical vertebra (c7, vertebra prominens) to the tip of the middle finger of both arms when they were stretched out sideways, using a tape measure [3]. After this, the participants received a standardised introduction to the test and demonstration of how to carry out the YBT-UQ correctly. Each participant then carried out three practice trials followed by three data-collection trials as recommend by Plisky [3]. Because of the amount of time needed for administering the YBT-UQ (i.e., three practice trials followed by three data-collection trials), two skilled examiners documented the reach distances in a group setting (i.e., examiner-to-pupil ratio: 1 to 5). Prior to commencing this study, both examiners completed a training session on the adequate execution of the YBT-UQ.

### Assessment of YBT-UQ performance

The YBT-UQ was carried out with the help of the YBT Kit (Functional Movement Systems®, Chatham, USA). The YBT Kit consists of a central element and three bars arranged in the shape of a Y. These three bars represent the reach directions: MD (to the side), IL (to the side, crossing over), and SL (towards the front). The bars are marked out in one-centimetre sections and fitted with three movable blocks. Based on the recommendations of Plisky [3], the participants’ task consisted of first assuming the one-arm push-up position with their left arm, and then pushing the relevant block as far as possible with their free (right) arm–first in the MD direction, then in the IL direction, and finally in the SL direction (Fig 1A–1D). Following this, the sequence was carried out with the right arm as supporting arm and the left as reaching arm. A trial was determined as failed and was repeated if any of the following criteria happened: 1) the subject did not maintain the one-arm push-up position at any point during the trial (i.e., touched down to the floor with the reach hand), 2) the subject did not maintain reach hand contact with the reach indicator (i.e., shoved the reach indicator), 3) the subject used the reach indicator for support (i.e., placed reach hand on top of the reach indicator), 4) the subject did not return the reach hand to the starting position under control, or 5) the subject lifted either foot off the ground. Readings for the absolute reach, in centimetres, of each arm in each direction were taken as dependent variables. Interrater reliability has previously been shown to be excellent (i.e., 0.98 ≤ ICC ≤ 1.00) for the YBT-UQ [13,14].

### Data and statistical analyses

For statistical analysis purposes, the readings for absolute maximum values (in cm) per reach direction were normalised (% AL) according to upper-extremity length (separately for the left and right arm). Further, a relative (% AL) composite score (CS) per arm was calculated, by dividing the sum of the absolute maximum reach distance (in cm) in each reach direction by three times the AL (in cm) and then multiplying the result by 100. The determination of the CS enables a general, i.e. direction-unspecific comparison of the test results while taking into account differing limb lengths [14,15]. Data are presented as group mean values ± standard deviations. Normal distribution of data across all sex by age groups was examined using the Shapiro Wilk test (*p* > 0.05). Multivariate analyses of variance (MANOVA) were performed to demonstrate whether the hypothesized differences in YBT-UQ performance between the four age groups and between girls and boy are statistically significant. If significant differences emerged, post-hoc tests were carried out. As a measurement of effect size, the partial eta squared (*η*_p_^2^) was also determined, and classified as small (0.02 ≤ *η*_p_^2^ ≤ 0.12), medium (0.13 ≤ *η*_p_^2^ ≤ 0.25), and large (*η*_p_^2^ ≥ 0.26) in accordance with Cohen [16]. Additionally, age- and sex-specific percentiles were compiled in tabular form (5^th^ to 95^th^ percentiles) with 95% confidence interval and in graphic form (10^th^, 50^th^, and 90^th^ percentiles). All statistical calculations were carried out using SPSS, Version 24.0 (SPSS Inc., Chicago, IL, USA).

## Results

### Age- and sex-specific YBT-UQ performance differences

Table 2 shows the mean values and standard deviations of normalised YBT-UQ (% AL) performance, subdivided according to age group and gender. Irrespective of reaching arm and reach direction, the MANOVA yielded a significant main effect of age in boys (all *p* < 0.001; range: 0.05 ≤ *η*_p_^2^ ≤ 0.14). For the MD direction, the post-hoc analysis revealed significantly greater values for the 14–15-year-olds compared with the 12–13-year-olds (left/right arm reach: *p* < 0.001) and the 16–17-year-olds (left/right arm reach: *p* < 0.001). For the IL direction, significantly larger values for the 14–15-year-olds emerged compared with the 12–13-year-olds (left/right arm reach: *p* < 0.001). For the SL direction, the analysis yielded significantly lower values for the 12–13-year-olds compared with the 10–11-year-olds (left arm reach: *p* = 0.039), the 14–15-year-olds (left/right arm reach: *p* < 0.001), and the 16–17-year-olds (left/right arm reach: *p* < 0.001). Finally, the CS for the 12–13-year-olds was significantly lower than that for the 10–11-year-olds (left arm reach: *p* = 0.015; right arm reach: *p* = 0.045), the 14–15-year-olds (left/right arm reach: *p* < 0.001), and the 16–17-year-olds (left/right arm reach: *p* < 0.001).

**Table 2:** Upper Quarter Y Balance test performance (% arm length) by age group and sex.

Age group	10–11 years (*N* = 56)		12–13 years (*N* = 175)		14–15 years (*N* = 218)		16–17 years (*N* = 216)	
Sex	f (*n* = 35)	m (*n* = 21)	f (*n* = 91)	m (*n* = 84)	f (*n* = 88)	m (*n* = 130)	f (*n* = 111)	m (*n* = 105)
*Right arm reach*								
Medial	100.7 (10.9)	100.1 (7.9)	95.7 (11.7)	92.9 (10.3)	98.3 (10.3)	104.1 (12.3)	96.6 (14.5)	96.5 (15.6)
Inferolateral	98.4 (17.4)	95.4 (14.5)	88.4 (13.7)	86.0 (16.3)	84.1 (13.1)	97.7 (15.8)	85.2 (15.8)	92.0 (20.6)
Superolateral	73.0 (15.2)	69.7 (17.1)	69.0 (12.5)	63.0 (12.9)	70.7 (14.4)	76.2 (13.5)	72.1 (15.5)	76.8 (16.0)
Composite score	90.7 (12.9)	88.4 (11.9)	84.5 (11.1)	80.5 (10.9)	84.3 (10.8)	92.6 (11.9)	84.6 (12.3)	88.4 (12.8)
*Left arm reach*								
Medial	98.2 (9.3)	99.5 (9.0)	95.8 (11.0)	93.1 (11.1)	96.9 (9.3)	103.7 (11.8)	94.9 (13.0)	95.8 (15.5)
Inferolateral	96.6 (14.6)	97.0 (14.4)	88.8 (14.7)	85.9 (14.5)	83.1 (12.5)	95.7 (15.8)	84.9 (15.6)	91.1 (21.0)
Superolateral	71.4 (12.8)	70.7 (15.9)	67.3 (12.0)	60.9 (13.0)	69.2 (14.2)	73.7 (14.0)	70.4 (15.4)	74.6 (16.4)
Composite score	88.7 (11.0)	89.1 (11.9)	84.0 (11.1)	80.1 (11.4)	83.1 (10.1)	91.1 (11.9)	83.4 (11.9)	87.2 (13.0)

Values are mean values and standard deviations in parentheses. f = female; m = male.

In girls, the MANOVA showed a significant main effect of age only for the IL direction (left arm reach: *p* < 0.001, *η*_p_^2^ = 0.07; right arm reach: *p* < 0.001, *η*_p_^2^ = 0.08) and the CS (right arm reach: *p* = 0.031, *η*_p_^2^ = 0.03). For the IL direction, the subsequent post-hoc analysis yielded significantly higher values for the 10–11-year-olds compared with the 12–13-year-olds (left arm reach: *p* = 0.043, right arm reach: *p* = 0.005), the 14–15-year-olds (left/right arm reach: *p* < 0.001), and the 16–17-year-olds (left/right arm reach: *p* < 0.001). Further, the CS for the 10–11-year-olds was also significantly greater than that for the 12–13-year-olds (right arm reach: *p* = 0.044), the 14–15-year-olds (right arm reach: *p* = 0.037), and the 16–17-year-olds (right arm reach: *p* = 0.045).

In 10–11-year-olds, there were no significant differences between girls and boys. However, significant sex differences were revealed in the case of the 12–13-year-olds with regard to the SL direction (left arm reach: *p* = 0.001, right arm reach: *p* = 0.002) and the CS (left arm reach: *p* = 0.026, right arm reach: *p* = 0.020) in favour of the females. Contrary, all values (*p* ≤ 0.021) for the 14–15-year-olds, irrespective of reaching arm and reach direction, came out to the males’ advantage. In the case of the 16–17-year-olds as well, the boys achieved significantly higher values than the girls for the IL direction (left arm reach: *p* = 0.014, right arm reach: *p* = 0.007), the SL direction (right arm reach: *p* = 0.029), and the CS (left arm reach: *p* = 0.026, right arm reach: *p* = 0.027).

### Age- and sex-specific YBT-UQ percentile values

**Fig 2:**
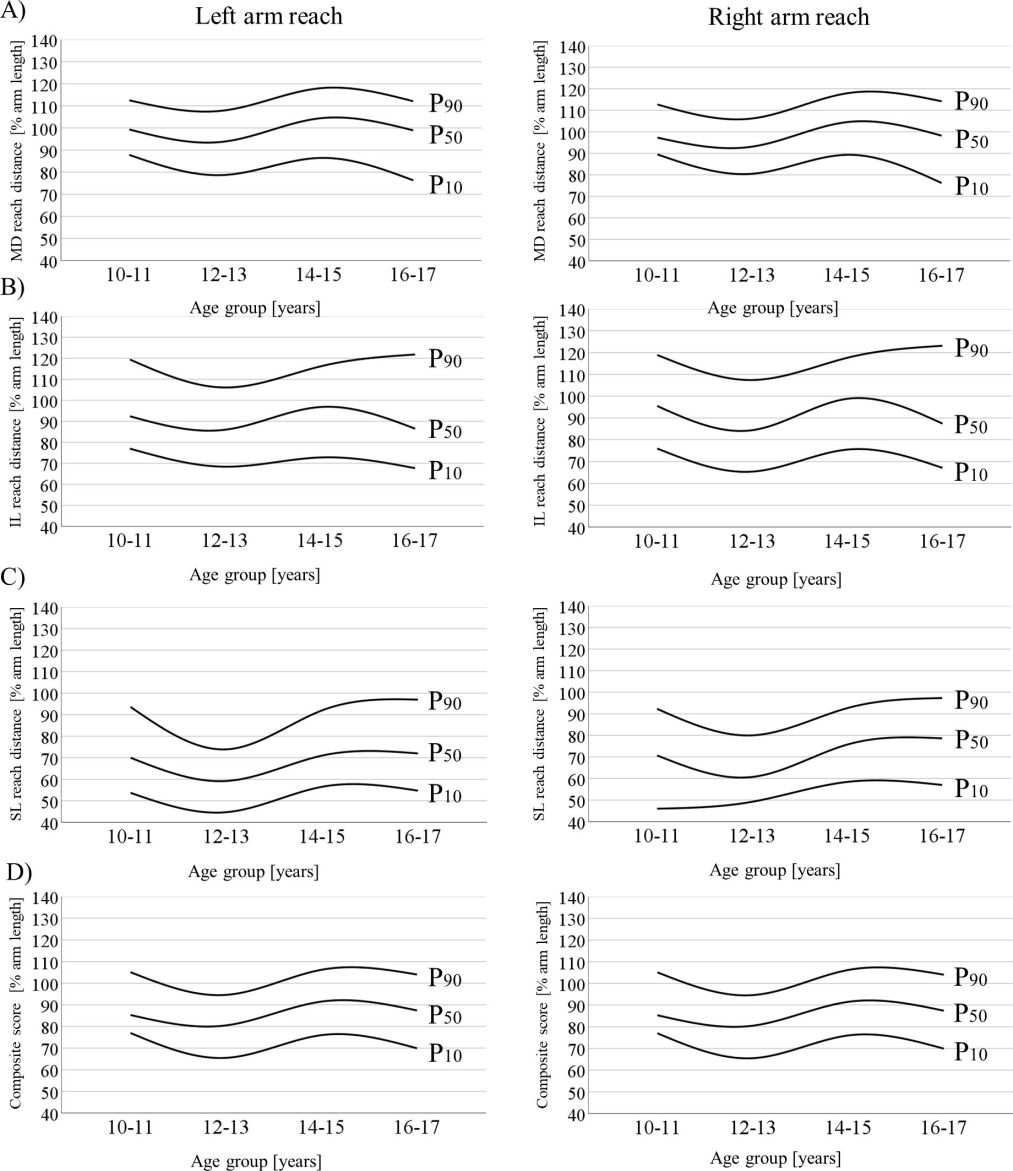
Smoothed curves for the 10^th^, 50^th^, and 90^th^ percentiles (% arm length) of the (A) medial, (B) inferolateral, and (C) superolateral reach directions as well as the (D) composite score during left and right arm reach in males aged 10 to 17 years. IL = inferolateral; MD = medial; SL = superolateral.

Table 3 (right arm reach), Table 4 (left arm reach) and Fig 2A–2D show the age-specific percentile values for the boys. Irrespective of reaching arm, we detected curvilinear developments. More specifically, the values for 10–11-year-olds were similar to those for the 14–15-year-olds, but higher than the values for the 12–13-year-olds and, in part (MD direction), also higher than the values for the 16–17-year-olds.

**Table 3:** Smoothed age-specific percentile values with 95% confidence interval for the normalized (% arm length) maximal right arm reach distances and the composite score in males (*n* = 340) aged 10 to 17 years.

Outcome	5^th^	10^th^	20^th^	30^th^	40^th^	50^th^	60^th^	70^th^	80^th^	90^th^	95^th^
Medial											
10–11 yrs (*n* = 21)	87.2 (86.2–89.6)	89.5 (86.9–94.5)	94.5 (93.6–95.5)	96.1 (96.1–96.2)	96.7 (96.1–96.9)	97.3 (97.0–97.9)	100.3 (99.3–102.0)	104.6 (103.3–106.1)	107.5 (105.8–108.9)	112.7 (106.1–116.2)	117.9 (111.4–120.7)
12–13 yrs (*n* = 84)	71.2 (69.4–76.6)	80.5 (76.8–81.5)	85.2 (83.6–85.7)	88.5 (87.6–88.8)	90.1 (90.0–90.6)	93.1 (92.3–93.8)	95.1 (94.9–95.8)	99.0 (98.7–99.2)	102.6 (100.6–102.7)	106.2 (105.3–106.8)	107.9 (106.7–111.9)
14–15 yrs (*n* = 130)	85.9 (77.0–87.5)	89.3 (88.3–90.1)	94.1 (92.7–94.4)	97.9 (97.1–98.4)	101.8 (101.0–102.2)	104.5 (104.2–105.3)	107.6 (107.1–107.9)	111.0 (110.4–111.4)	113.9 (113.2–114.7)	117.9 117.7–120.5	124.1 (121.6–128.1)
16–17 yrs (*n* = 105)	65.3 (59.3–69.4)	76.2 (71.2–77.3)	84.5 (82.8–86.1)	90.2 (89.4–91.8)	95.3 (94.6–95.9)	98.2 (97.7–98.4)	101.2 (100.2–101.6)	105.0 (104.1–105.8)	108.5 (107.9–109.3)	114.2 (113.3–117.1)	121.6 (117.8–125.3)
Inferolateral											
10–11 yrs (*n* = 21)	68.3 (63.9–78.9)	76.0 (72.5–82.6	82.4 (82.1–82.9)	87.9 (87.3–88.4)	90.6 (88.6–91.6)	95.5 (93.0–96.3)	98.9 (98.6–99.1)	100.9 (99.3–102.2)	105.5 (102.9–107.6)	118.9 (107.2–125.1)	126.9 (117.4–130.9)
12–13 yrs (*n* = 84)	61.1 (60.3–65.5)	65.4 (63.6–67.3)	70.9 (70.1–72.2)	76.3 (75.7–77.7)	81.4 (80.7–82.1)	84.4 (84.0–85.4)	89.2 (88.2–90.4)	94.3 (94.0–95.9)	98.8 (97.8–100.3)	107.4 (104.2–112.0)	120.3 (113.4–123.0)
14–15 yrs (*n* = 130)	71.2 (65.9–72.4)	75.5 (74.3–77.2)	82.8 (81.6–83.2)	86.9 (86.4–89.4)	95.8 (94.4–95.9)	98.8 (98.0–98.9)	102.6 (101.9–103.8)	108.3 (107.8–109.6)	114.4 (112.8–114.6)	117.6 (117.3–118.6)	120.3 (119.1–121.7)
16–17 yrs (*n* = 105)	63.5 (59.8–66.1)	67.1 (65.9–68.5)	75.8 (73.9–76.5)	79.8 (79.2–80.6)	84.0 (83.7–84.3)	87.4 (86.6–87.8)	92.2 (91.6–93.0)	98.1 (96.3–100.8)	112.0 (111.1–114.5)	123.1 (120.4–126.9)	131.1 (128.7–138.7)
Superolateral											
10–11 yrs (*n* = 21)	36.2 (31.1–48.1)	46.0 (39.1–59.0)	55.0 (53.5–56.9)	58.2 (55.6–60.3)	59.6 (59.0–60.6)	70.7 (68.1–72.2)	74.7 (71.4–80.9)	82.8 (77.4–89.4)	89.6 (86.1–92.5)	92.3 (91.3–92.9)	93.3 (92.1–93.9)
12–13 yrs (*n* = 84)	44.6 (40.7–46.6)	49.2 (47.2–49.9)	52.2 (51.7–52.9)	55.2 (54.7–55.7)	56.8 (56.5–57.8)	60.7 (59.7–61.4)	65.5 (64.2–66.2)	69.8 (69.4–70.2)	74.4 (73.2–75.4)	80.0 (78.9–83.7)	87.3 (83.3–93.4)
14–15 yrs (*n* = 130)	51.8 (49.9–55.3)	58.4 (56.7–59.7)	65.6 (63.9–66.1)	69.9 (69.1–70.3)	73.0 (72.4–73.2)	75.7 (75.3–76.5)	80.0 (79.2–80.2)	82.2 (82.1–83.6)	87.1 (86.8–88.5)	92.6 (91.9–95.0)	99.1 (97.2–104.5)
16–17 yrs (*n* = 105)	52.1 (47.3–54.0)	57.0 (54.1–57.5)	61.7 (60.3–61.9)	66.9 (66.2–68.5)	71.0 (70.7–72.2)	78.6 (76.7–79.0)	81.1 (80.6–81.2)	84.3 (83.6–85.3)	90.1 (89.7–92.4)	97.3 (96.6–101.3)	105.8 (102.4–109.4)
Composite score											
10–11 yrs (*n* = 21)	69.9 (66.8–77.2)	74.7 (74.1–74.9)	75.7 (73.5–78.5)	80.4 (80.0–80.7)	81.6 (81.1–82.0)	88.5 (82.0–92.8)	94.7 (94.3–95.6)	95.9 (95.8–96.0)	98.8 (94.4–102.4)	105.2 (99.8–108.1)	112.4 (102.7–116.6)
12–13 yrs (*n* = 84)	64.4 (59.3–66.4)	67.2 (66.4–67.8)	71.5 (69.8–71.7)	74.8 (73.8–75.4)	77.4 (76.6–77.6)	79.5 (78.7–80.1)	83.0 (82.4–83.7)	86.5 (86.0–87.2)	90.0 (89.0–91.4)	95.0 (93.9–96.2)	100.5 (96.8–103.8)
14–15 yrs (*n* = 130)	73.4 (69.0–74.4)	77.1 (75.5–77.5)	81.8 (80.3–82.0)	85.3 (84.8–86.3)	89.9 (89.1–90.0)	93.3 (93.0–94.0)	96.3 (96.2–97.2)	100.4 (99.6–101.0)	103.3 (103.0–104.0)	106.8 (106.7–108.2)	110.3 (109.1–114.0)
16–17 yrs (*n* = 105)	67.0 (64.0–69.4)	72.1 (70.0–73.1)	77.0 (76.4–78.0)	80.6 (80.1–81.8)	85.3 (84.9–85.6)	88.0 (87.6–88.6)	91.2 (90.8–91.5)	94.0 (93.2–94.8)	100.6 (99.1–101.5)	107.0 (105.8–108.0)	110.9 (108.9–113.7)

**Table 4:** Smoothed age-specific percentile values with 95% confidence interval for the normalized (% arm length) maximal left arm reach distances and the composite score in males (n = 340) aged 10 to 17 years.

Outcome	5^th^	10^th^	20^th^	30^th^	40^th^	50^th^	60^th^	70^th^	80^th^	90^th^	95^th^
Medial											
10–11 yrs (*n* = 21)	86.8 (86.3–88.1)	87.8 (87.3–88.8)	90.8 (89.8–92.0)	93.8 (93.7–93.9)	95.8 (93.3–97.1)	99.3 (97.1–100.5)	102.1 (102.0–102.4)	102.9 (101.2–105.1)	109.0 (105.3–111.9)	112.5 (111.7–113.0)	118.3 (109.6–121.9)
12–13 yrs (*n* = 84)	74.4 (68.7–77.8)	78.7 (77.5–79.7)	81.8 (81.2–84.0)	87.4 (87.2–87.9)	91.6 (90.2–91.8)	93.8 (93.1–94.0)	95.2 (94.9–96.3)	98.7 (98.0–99.8)	104.1 (102.7–104.8)	107.9 (107.2–109.3)	110.7 (109.6–112.6)
14–15 yrs (*n* = 130)	80.9 (77.5–83.9)	86.4 (85.7–89.0)	94.1 (93.4–94.7)	98.9 (97.6–99.1)	101.8 (100.8–101.9)	104.3 (103.7–104.7)	108.4 (107.5–108.9)	110.6 (110.3–111.2)	113.2 (112.6–113.5)	117.9 (117.2–119.8)	122.0 (121.0–124.7)
16–17 yrs (*n* = 105)	66.6 (57.9–69.7)	76.3 (71.1–77.3)	84.1 (81.8–84.5)	89.5 (88.4–90.2)	94.7 (94.2–95.3)	98.9 (97.7–99.0)	101.2 (100.8–102.0)	104.9 (104.5–105.8)	108.0 (107.8–108.6)	112.1 (111.0–115.3)	117.8 (116.2–123.4)
Inferolateral											
10–11 yrs (*n* = 21)	75.8 (75.1–77.4)	77.7 (74.6–83.7)	84.8 (82.9–87.1)	87.0 (86.3–87.6)	88.7 (87.5–89.3)	92.4 (89.5–95.0)	101.3 (96.8–109.8)	107.9 (107.3–108.5)	113.9 (112.9–114.8)	119.4 (112.1–120.8)	120.9 (120.4–121.1)
12–13 yrs (*n* = 84)	62.4 (58.9–65.6)	68.4 (66.2–70.2)	73.9 (73.1–74.4)	78.0 (77.3–78.7)	81.4 (80.6–82.3)	86.0 (85.3–86.8)	89.2 (88.6–89.4)	90.7 (90.2–91.2)	95.7 (94.4–97.6)	106.1 (103.8–109.4)	113.7 (109.6–120.6)
14–15 yrs (*n* = 130)	67.4 (65.7–69.7)	72.8 (70.7–74.0)	81.8 (80.9–82.1)	85.5 (84.8–87.1)	91.9 (91.0–82.3)	96.8 (95.9–97.7)	101.6 (101.2–102.4)	105.3 (105.1–106.6)	110.6 (109.7–111.4)	116.1 (115.3–117.1)	119.3 (118.2–122.8)
16–17 yrs (*n* = 105)	63.7 (59.4–65.3)	67.7 (66.4–69.1)	72.5 (72.0–73.2)	78.0 (76.7–79.0)	81.8 (81.2–83.7)	86.5 (85.9–87.4)	90.8 (90.1–92.3)	97.9 (96.3–100.8)	111.4 (109.5–113.1)	121.8 (120.0–126.6)	134.0 (129.0–139.0)
Superolateral											
10–11 yrs (*n* = 21)	40.6 (32.2–60.4)	53.7 (53.2–54.5	56.1 (54.6–57.8)	57.4 (57.1–57.6)	62.2 (61.4–62.6)	70.0 (67.7–72.1)	78.8 (77.3–81.8)	83.3 (80.9–86.2)	85.3 (85.0–85.4)	93.6 (85.6–97.9)	97.3 (93.6–98.8)
12–13 yrs (*n* = 84)	42.4 (39.6–43.5)	44.7 (43.7–46.5)	50.6 (49.7–51.1)	53.9 (53.6–54.5)	57.5 (56.4–58.2)	59.2 (59.1–60.0)	62.8 (62.1–63.9)	67.5 (66.3–67.8)	68.8 (68.5–70.6)	73.9 (73.6–79.1)	84.1 (77.7–97.1)
14–15 yrs (*n* = 130)	49.0 (46.5–52.8)	56.5 (54.6–58.0)	62.8 (61.9–63.3)	65.9 (65.5–66.4)	68.8 (68.5–69.1)	71.0 (70.7–72.6)	77.2 (76.3–77.9)	80.9 (80.6–81.6)	86.3 (85.4–87.7)	92.0 (91.5–94.4)	97.8 (95.4–101.3)
16–17 yrs (*n* = 105)	51.1 (44.1–52.7)	54.7 (53.2–55.6)	59.6 (58.9–60.8)	64.7 (64.0–65.6)	68.7 (68.0–68.8)	72.0 (71.6–73.2)	77.8 (76.5–78.9)	82.3 (81.8–84.4)	91.4 (89.8–92.4)	97.0 (96.0–100.2)	104.0 (101.0–107.5)
Composite score											
10–11 yrs (*n* = 21)	69.0 (64.8–80.8)	77.0 (76.1–78.6)	78.7 (77.9–79.7)	79.7 (79.2–80.1)	80.6 (79.7–81.1)	85.3 (83.1–89.1)	94.2 (90.9–100.4)	98.3 (98.0–98.6)	103.0 (101.4–104.3)	105.1 (103.4–106.0)	109.0 (103.5–111.3)
12–13 yrs (*n* = 84)	62.9 (58.3–64.5)	65.5 (63.8–66.8)	70.4 (69.7–71.2)	72.9 (72.5–73.1)	76.8 (75.9–78.2)	80.5 (80.2–80.9)	82.1 (81.7–82.7)	84.9 (84.5–85.4)	89.1 (87.7–89.8)	94.6 (93.0–96.0)	101.0 (97.1–107.4)
14–15 yrs (*n* = 130)	71.6 (68.6–72.7)	76.0 (73.6–76.2)	79.0 (78.6–80.2)	83.5 (82.7–84.4)	88.7 (87.7–89.1)	91.5 (91.0–92.2)	95.3 (94.8–96.0)	99.4 (98.3–99.5)	101.3 (101.1–102.8)	106.2 (105.9–107.5)	109.6 (108.1–111.9)
16–17 yrs (*n* = 105)	67.1 (63.3–68.5)	69.9 (69.0–71.1)	74.9 (73.7–75.5)	79.4 (78.1–79.7)	83.9 (82.9–84.4)	87.4 (86.6–88.1)	90.9 (90.3–91.3)	95.4 (93.8–95.5)	99.0 (98.1–99.8)	104.0 (102.9–105.5)	108.6 (106.4–113.4)

**Fig 3:**
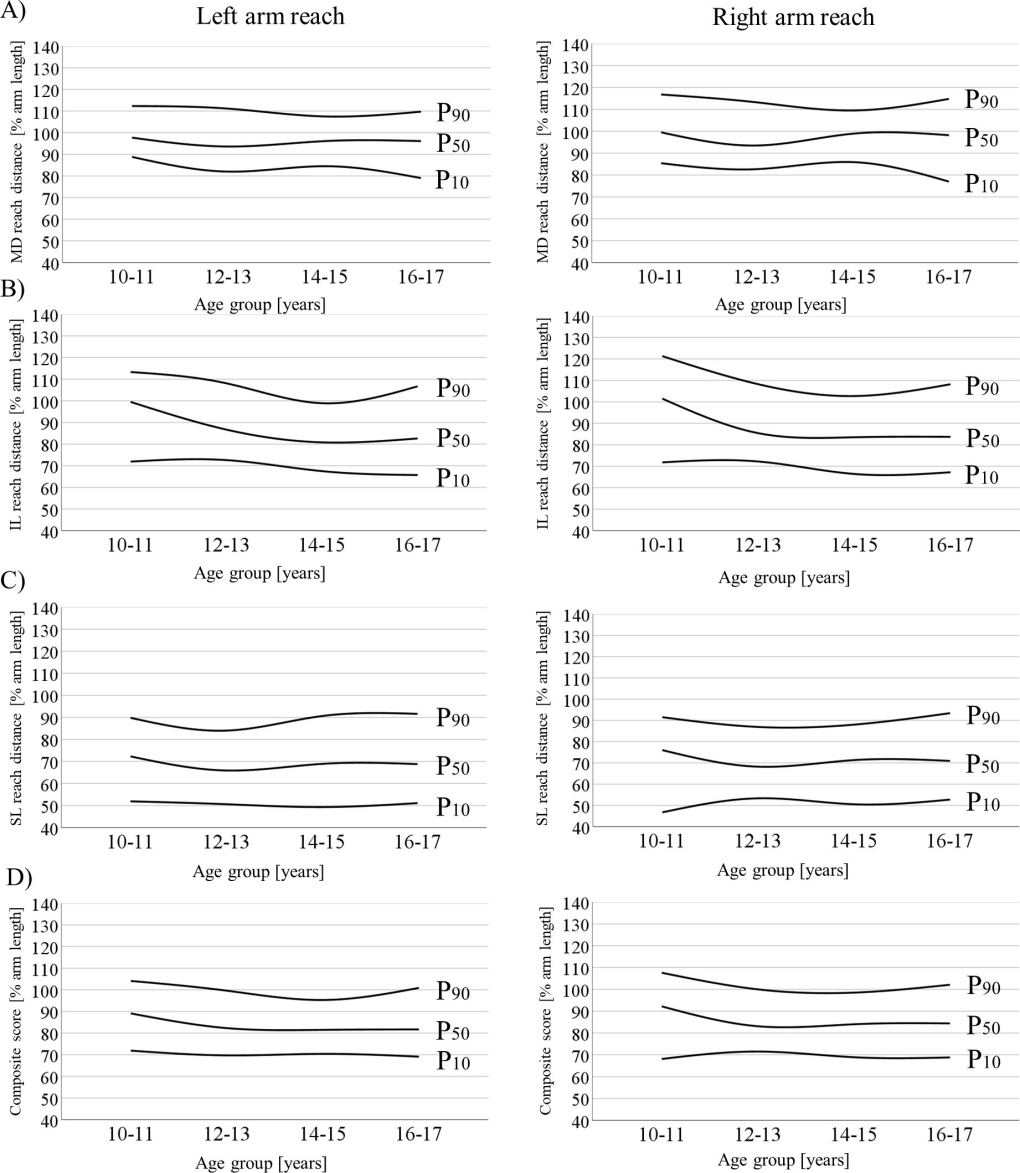
Smoothed curves for the 10^th^, 50^th^, and 90^th^ percentiles (% arm length) of the (A) medial, (B) inferolateral, and (C) superolateral reach directions as well as the (D) composite score during left and right arm reach in females aged 10 to 17 years. IL = inferolateral; MD = medial; SL = superolateral.

Table 5 (right arm reach), Table 6 (left arm reach) and Fig 3A–3D illustrate the age-specific percentile values for the girls. In contrast to the boys, these displayed less strongly pronounced curvilinear developments. Particularly noticeable are the higher values in 10–11-year-olds, which decrease in 12–13- and 14–15-year-olds and finally stabilise (MD and SL direction) or increase slightly again (IL direction) in 16–17-year-olds.

**Table 5:** Smoothed age-specific percentile values with 95% confidence interval for the normalized (% arm length) maximal right arm reach distances and the composite score in females (*n* = 325) aged 10 to 17 years.

Outcome	5^th^	10^th^	20^th^	30^th^	40^th^	50^th^	60^th^	70^th^	80^th^	90^th^	95^th^
Medial											
10–11 yrs (*n* = 35)	81.9 (74.9–87.2)	85.4 (83.1–89.5)	93.3 (91.8–94.5)	95.4 (94.8–95.8)	98.0 (96.8–98.9)	99.5 (99.3–99.6)	101.1 (100.1–101.2)	105.3 (104.5–106.9)	111.2 (110.2–112.8)	116.8 (113.5–121.1)	122.4 (118.7–124.7)
12–13 yrs (*n* = 91)	79.2 (75.3–81.5)	82.7 (81.2–83.3)	84.9 (84.5–85.6)	88.1 (87.5–88.7)	91.6 (91.2–92.1)	93.5 (93.4–94.4)	96.8 (96.4–98.2)	101.2 (100.1–101.9)	105.4 (104.8–107.3)	113.2 (111.6–115.1)	117.6 (115.2–121.5)
14–15 yrs (*n* = 88)	83.4 (75.8–86.0)	85.9 (85.7–86.8)	89.1 (88.5–90.5)	92.6 (92.5–93.2)	95.4 (95.0–96.1)	98.9 (98.0–99.8)	101.5 (100.9–101.9)	103.4 (102.7–103.4)	105.5 (104.7–106.0)	109.5 108.6–112.9)	117.4 (114.5–121.9)
16–17 yrs (*n* = 111)	70.7 (68.6–73.9)	77.0 (74.7–77.9)	83.0 (82.5–83.9)	88.2 (87.3–88.9)	93.0 (92.2–93.5)	98.2 (96.8–98.9)	101.3 (100.5–101.5)	104.9 (104.4–105.9)	109.4 (108.7–109.9)	114.8 (113.8–116.4)	118.3 (116.4–124.4)
Inferolateral											
10–11 yrs (*n* = 35)	68.1 (65.6–71.6)	71.8 (69.3–74.0)	82.7 (78.8–85.4)	89.3 (86.9–91.6)	94.1 (92.4–95.4)	101.5 (99.1–102.3)	103.2 (101.7–105.3)	110.6 (107.2–112.3)	114.2 (112.5–117.3)	121.3 (118.9–123.9)	126.3 (119.8–134.7)
12–13 yrs (*n* = 91)	69.5 (67.8–70.6)	72.2 (70.6–72.6)	76.8 (75.2–77.3)	79.4 (79.1–80.4)	82.7 (82.3–83.3)	85.4 (84.8–85.7)	89.0 (87.4–90.6)	95.9 (94.6–96.7)	101.6 (100.3–103.2)	108.2 (107.5–109.8)	112.4 (110.3–118.4)
14–15 yrs (*n* = 88)	62.1 (60.1–64.3)	66.4 (64.9–68.1)	72.1 (71.2–72.6)	77.0 (75.4–78.1)	79.9 (79.5–81.5)	83.5 (83.3–83.7)	85.6 (84.7–87.0)	91.8 (91.1–92.4)	96.1 (95.1–97.1)	102.7 (101.1–104.5)	107.7 (105.6–109.7)
16–17 yrs (*n* = 111)	63.1 (57.5–65.0)	67.2 (65.3–67.8)	70.9 (69.9–71.7)	74.9 (74.0–75.5)	78.1 (78.0–79.6)	83.7 (82.6–84.5)	88.3 (87.8–89.1)	92.9 (91.8–93.6)	99.5 (98.3–101.1)	108.2 (106.4–109.3)	112.2 (108.8–113.5)
Superolateral											
10–11 yrs (*n* = 35)	43.7 (42.4–45.3)	46.7 (43.8–50.4)	55.8 (53.4–60.2)	68.1 (66.7–69.9)	73.5 (72.1–74.2)	76.0 (74.3–77.1)	80.1 (79.1–80.8)	81.3 (80.7–82.7)	87.7 (83.8–89.9)	91.5 (90.6–92.3)	92.8 (91.1–94.6)
12–13 yrs (*n* = 91)	50.1 (46.9–52.4)	53.3 (52.1–54.1)	57.8 (57.2–58.0)	60.6 (60.0–61.8)	64.8 (64.0–65.2)	68.2 (67.5–68.6)	70.4 (69.8–71.8)	76.3 (74.8–77.1)	81.2 (80.1–81.9)	86.9 (86.2–89.6)	91.8 (89.6–93.3)
14–15 yrs (*n* = 88)	44.6 (42.1–47.1)	50.5 (48.1–51.9)	58.8 (56.0–60.0)	64.0 (63.5–64.4)	67.1 (66.4–67.9)	71.3 (69.9–71.6)	74.9 (73.5–75.3)	80.1 (78.0–80.7)	84.0 (83.3–84.5)	88.0 (86.7–89.3)	92.4 (89.3–100.2)
16–17 yrs (*n* = 111)	49.3 (44.1–51.6)	52.7 (51.7–53.4)	57.5 (56.6–58.8)	62.6 (62.3–64.1)	67.4 (67.1–68.2)	70.9 (70.4–71.5)	75.1 (74.5–76.1)	79.5 (79.1–81.0)	85.3 (84.2–86.7)	93.4 (92.3–95.5)	98.2 (96.3–104.9)
Composite score											
10–11 yrs (*n* = 35)	66.1 (65.3–68.1)	68.1 (66.7–69.1)	78.3 (76.4–84.3)	87.1 (86.8–87.8)	89.8 (88.2–91.4)	92.2 (92.1–92.5)	94.2 (93.6–94.6)	97.1 (96.1–98.1)	101.2 (100.3–102.0)	107.6 (105.8–109.4)	110.6 (107.1–115.6)
12–13 yrs (*n* = 91)	68.5 (66.0–69.8)	71.5 (70.1–72.0)	74.6 (74.1–75.0)	76.4 (76.3–77.5)	79.7 (78.9–80.0)	83.1 (82.3–83.5)	86.3 (85.4–87.1)	89.9 (89.6–91.4)	96.9 (95.9–97.5)	100.0 (99.2–102.1)	104.9 (102.4–106.4)
14–15 yrs (*n* = 88)	64.3 (62.6–65.7)	68.9 (66.3–70.5)	76.6 (74.7–76.9)	78.6 (78.2–78.7)	80.4 (79.8–81.1)	84.0 (83.5–84.7)	87.9 (87.7–88.1)	90.8 (89.9–91.7)	94.1 (93.8–94.7)	98.5 (97.1–99.8)	101.9 (100.6–104.0)
16–17 yrs (*n* = 111)	65.9 (60.2–67.1)	68.8 (67.6–70.4)	74.6 (73.7–74.8)	78.4 (77.2–78.7)	80.2 (80.1–80.7)	84.4 (83.0–85.0)	88.1 (87.3–88.4)	90.9 (90.1–91.3)	94.3 (93.6–96.0)	102.1 (101.2–103.0)	104.8 (103.9–108.1)

**Table 6:** Smoothed age-specific percentile values with 95% confidence interval for the normalized (% arm length) maximal left arm reach distances and the composite score in females (*n* = 325) aged 10 to 17 years.

Outcome	5^th^	10^th^	20^th^	30^th^	40^th^	50^th^	60^th^	70^th^	80^th^	90^th^	95^th^
Medial											
10–11 yrs (*n* = 35)	78.3 (73.1–88.9)	88.8 (81.6–91.3)	90.6 (90.2–91.7)	93.3 (92.4–93.7)	95.9 (94.8–96.5)	97.7 (97.2–98.6)	99.8 (99.2–100.4)	102.6 (101.6–103.5)	104.8 (103.6–107.6)	112.3 (109.0–116.1)	116.5 (114.7–117.3)
12–13 yrs (*n* = 91)	80.3 (76.5–81.5)	82.0 (81.3–83.4)	86.7 (86.2–87.6)	89.3 (89.0–89.8)	91.4 (91.0–92.0)	93.6 (93.5–94.9)	97.6 (96.6–98.1)	100.4 (99.4–101.5)	107.0 (105.6–107.4)	111.1 (110.3–113.9)	116.5 (113.7–119.0)
14–15 yrs (*n* = 88)	82.2 (79.7–83.4)	84.5 (83.8–85.4)	87.6 (87.5–88.5)	92.2 (91.6–92.6)	93.9 (93.5–94.3)	96.1 (95.7–97.0)	100.0 (98.7–100.3)	102.4 (101.5–102.6)	105.7 (104.6–106.0)	107.5 (107.3–109.8)	112.9 (110.8–116.9)
16–17 yrs (*n* = 111)	72.3 (68.1–75.3)	79.0 (76.4–79.6)	82.9 (81.9–83.8)	87.8 (87.3–89.2)	92.5 (92.1–93.6)	96.1 (95.7–96.9)	98.2 (98.0–98.5)	101.4 (100.3–101.9)	106.1 (105.0–106.3)	109.7 (109.3–111.9)	115.6 (112.5–121.2)
Inferolateral											
10–11 yrs (*n* = 35)	67.7 (66.6–70.5)	71.9 (68.6–73.7)	85.2 (80.4–87.1)	93.3 (92.8–93.8)	94.8 (93.9–96.0)	99.5 (96.6–100.8)	101.3 (100.8–101.7)	104.9 (104.0–106.5)	108.9 (107.7–110.7)	113.3 (111.6–118.1)	120.6 (113.6–125.0)
12–13 yrs (*n* = 91)	67.1 (63.4–69.7)	72.6 (70.0–72.9)	76.0 (75.4–76.4)	79.1 (78.6–79.5)	83.2 (81.9–83.6)	86.7 (85.0–88.2)	93.6 (92.8–93.9)	96.0 (95.2–96.4)	100.0 (99.1–101.4)	108.1 (106.7–112.4)	117.0 (112.4–122.2)
14–15 yrs (*n* = 88)	64.1 (62.9–64.7)	67.5 (65.7–68.9)	72.6 (72.0–73.6)	76.3 (76.2–76.5)	78.3 (77.9–78.8)	80.8 (80.1–81.7)	84.6 (83.6–85.0)	89.0 (87.9–90.1)	94.4 (93.9–95.2)	98.9 (97.8–101.1)	105.6 (102.5–113.7)
16–17 yrs (*n* = 111)	62.0 (60.0–63.6)	65.7 (64.0–66.1)	71.9 (70.9–72.2)	75.5 (74.3–76.0)	78.0 (77.1–79.7)	82.6 (82.2–83.3)	86.6 (85.6–87.9)	92.9 (91.7–94.3)	97.9 (97.2–100.7)	106.7 (105.2–108.5)	115.1 (109.9–118.8)
Superolateral											
10–11 yrs (*n* = 35)	50.0 (45.7–53.1)	51.9 (51.1–52.7)	56.4 (53.4–62.3)	67.0 (65.2–68.8)	70.3 (69.2–71.2)	72.3 (71.9–73.8)	74.9 (73.7–75.7)	78.4 (74.9–82.0)	83.1 (82.8–83.4)	89.8 (88.0–91.1)	91.1 (90.1–91.6)
12–13 yrs (*n* = 91)	47.1 (46.0–49.3)	50.6 (49.1–52.0)	55.7 (54.5–55.9)	61.1 (59.1–61.7)	64.0 (63.5–64.4)	65.9 (65.6–67.1)	71.1 (69.7–72.0)	75.2 (74.3–76.2)	79.2 (78.5–79.5)	84.0 (82.6–85.3)	87.6 (85.6–89.5)
14–15 yrs (*n* = 88)	42.9 (41.3–45.2)	49.3 (46.4–51.8)	58.3 (57.7–58.9)	61.1 (60.8–61.5)	65.2 (64.4–65.7)	68.9 (67.8–69.2)	73.3 (71.6–73.7)	76.8 (75.7–77.9)	81.2 (80.9–83.3)	90.6 (87.3–91.5)	93.8 (91.8–97.2)
16–17 yrs (*n* = 111)	49.3 (45.7–50.3)	51.1 (50.8–52.0)	56.2 (55.4–57.6)	61.0 (60.2–61.3)	65.5 (64.0–66.6)	68.8 (68.6–69.1)	72.3 (71.6–73.3)	77.0 (76.2–77.5)	82.1 (80.8–84.6)	91.6 (91.0–95.1)	101.1 (96.0–105.5)
Composite score											
10–11 yrs (*n* = 35)	65.9 (62.0–72.2)	71.9 (68.1–74.0)	79.9 (75.5–82.3)	85.5 (85.1–85.9)	88.5 (88.0–89.0)	89.1 (88.8–89.9)	90.9 (90.6–91.3)	92.8 (92.6–93.0)	97.6 (95.2–101.6)	104.1 (102.1–106.9)	107.9 (104.9–109.4)
12–13 yrs (*n* = 91)	65.4 (64.9–68.4)	69.7 (68.5–70.5)	73.5 (72.9–74.2)	76.7 (76.2–77.1)	80.3 (79.5–80.7)	82.3 (81.8–83.1)	85.6 (85.1–85.9)	92.0 (90.4–92.3)	94.5 (94.0–95.9)	99.6 (99.1–100.8)	102.8 (101.4–105.2)
14–15 yrs (*n* = 88)	68.3 (66.1–69.5)	70.4 (70.0–71.2)	73.3 (72.6–74.3)	76.6 (76.2–77.0)	78.5 (78.2–78.9)	81.5 (80.5–82.9)	85.9 (85.2–86.7)	90.3 (89.5–90.5)	92.8 (91.9–93.2)	95.3 (95.1–97.0)	101.2 (98.1–104.5)
16–17 yrs (*n* = 111)	65.4 (62.8–67.2)	69.1 (67.7–69.8)	72.2 (71.8–73.3)	76.5 (76.1–77.0)	79.8 (79.3–80.1)	81.7 (81.4–82.6)	85.3 (84.5–85.4)	88.1 (87.4–89.4)	94.2 (92.7–94.5)	100.9 (99.1–102.8)	107.4 (103.7–108.9)

## Discussion

The objective was to investigate the YBT-UQ performance of boys and girls aged between 10 and 17 years and to provide age- and sex-specific normative values. The key findings may be summarised as follows. Among other age-specific differences, the 14–15-year-old boys achieved better values (e.g., in the MD direction) compared with the younger (12–13-year-olds) and older (16–17-year-olds) ones. In the case of the girls, better YBT-UQ performance in 10–11-year-olds compared with the higher age groups was only observed for the IL direction and the CS. With regard to sex-specific differences, better values in favour of girls emerged in 12–13-year-olds (SL direction and CS), and in favour of boys in 14–15-year-olds (all reach directions) and in 16–17-year-olds (IL and SL direction and CS). Further, curvilinear developments were observed with regard to the 10^th^, 50^th^, and 90^th^ percentiles, and were less strongly marked in girls than in boys.

### Age and sex differences in YBT-UQ performance

The first hypothesis made was that YBT-UQ performance improves with increasing age. Despite the fact that the 14–15-year-old boys showed better values (MD and IL direction) than the 12–13-year-old boys, they also achieved better values (MD direction) than the 16–17-year-old boys. In addition, although the 12–13-year-old boys achieved lower values (SL direction) than the 14–15- and 16–17-year-old boys, they also achieved lower values than the 10–11-year-old boys. In the case of the girls, it is true that a significant age-specific difference emerged for one of the three reach directions only (IL direction) and for the CS. However, the 10–11-year-old girls achieved better performance than the older age groups. A possible explanation for our findings might be given by considering the growth and maturation processes taking place in childhood and adolescence. These do not happen in a linear fashion but in a multi-tiered progression [17], and are accompanied by changes in performance level that are varyingly pronounced even over equal time periods [18]. Moreover, the growth and maturation processes of boys and girls proceed differently [19,20]. This may clarify the second of our hypotheses, sex-specific differences. We observed better performance, on the one hand, to the girls’ advantage in 12–13-year-olds (SL direction and CS) and, on the other hand, to the boys’ advantage among both 14–15-year-olds (all reach directions) and 16–17-year-olds (IL and SL direction and CS). The fact the girls performed better during an earlier age segment, and the boys better during a later one, could reflect the onset of growth and maturation processes at differing points in time. For example, Marshall and Tanner [21,22] were able to show that the ‘growth spurt’ already takes place around the age of 12 in girls, and not until around the age of 14 in boys. The observed differences between younger and older individuals as well as between girls and boys indicate that age- and sex-specific normative values are necessary for an adequate classification of YBT-UQ performance in healthy youth.

### Age- and sex-specific normative values for the YBT-UQ

Since there have, so far, been no studies investigating age- and sex-specific reference values for the YBT-UQ in children and adolescents, it has only been possible to compare the results obtained with the findings of studies among older persons. Borms and Cools [10] investigated 18–25-year-olds (among others) and reported the following YBT-UQ performance values: 102–104% AL (male) and 96–102% AL (female) for the MD direction; 90–94% AL (male) and 83–86% AL (female) for the IL directions; 68–73% AL (male) and 63–96% AL (female) for the SL direction; and 87–90% AL (male) and 81–85% AL (female) for the CS. A comparison of these values with the 50^th^ percentile values of the present study revealed a roughly equal and, in part, also lower or higher performance level. More specifically, the young participants in the present study achieved YBT-UQ performance values of: 93.1–104.5% AL (male) and 93.5–99.5% (female) for the MD direction; 84.4–98.8% AL (male) and 80.8–101.5% AL (female) for the IL direction; 59.2–78.6% AL (male) and 65.9–76.0% AL (female) for the SL direction; and 79.5–93.3% AL (male) and 81.5–92.2% AL (female) for the CS.

From a practical perspective, the age- and sex-specific normative values for the YBT-UQ obtained here can be used by teachers, coaches, and therapists to classify the performance level achieved by a child/adolescent. In a subsequent stage, specifically customised interventions could then be recommended on the basis of these, depending on the percentile range. For example, one might recommend a programme for talent development (e.g., overhead sports) to a child achieving good results, and a programme that boosts motor ability to a child achieving poor ones. Moreover, the percentile data can be used by clinicians to assist in rehabilitation programs in order to identify the transition from poor (10^th^ percentile) over moderate (50^th^ percentile) to good (90^th^ percentile) performance values. In addition, the data can help to decide whether a return to sport can take place after injury treatment or whether there is still an increased injury risk [8,9]. Lastly, the data can assist coaches in overhead sports to derive associations with athletic performance and to differentiate between athletes with different performance levels [6,7].

## Conclusions

We investigated the YBT-UQ performance of 10–17-year-old boys and girls and provides age- and sex-specific reference values. Contrary to our hypothesis, neither the boys nor the girls showed a clear improvement in YBT-UQ performance with increasing age. For example, the 14–15-year-old boys achieved better values than the 16–17-year-olds for the MD direction, and the 10–11-year-old girls achieved better results for the IL direction and the CS than all the other older age groups. With respect to sex differences, too, an uneven picture emerged, with better performance in the 12–13-year-old girls on the one hand (SL direction and CS), and better performance in the 14–15-year-old boys (all reach directions) and 16–17-year-old boys (IL and SL direction of and CS) on the other hand. In addition, curvilinear developments were observed for the 10^th^, 50^th^, and 90^th^ percentiles, and were more strongly pronounced among the boys compared to the girls. Thus, the generated normative values can be used by practitioners (e.g., P.E. teachers, trainers, therapists) to classify the individual level of YBT-UQ performance achieved depending on age and gender.

## Supporting information

S1 Data (SAV)
